# Networked Chemoreceptors Benefit Bacterial Chemotaxis Performance

**DOI:** 10.1128/mBio.01824-16

**Published:** 2016-12-20

**Authors:** Vered Frank, Germán E. Piñas, Harel Cohen, John S. Parkinson, Ady Vaknin

**Affiliations:** a The Racah Institute of Physics, the Hebrew University, Jerusalem, Israel; b Biology Department, University of Utah, Salt Lake City, Utah, USA

## Abstract

Motile bacteria use large receptor arrays to detect and follow chemical gradients in their environment. Extended receptor arrays, composed of networked signaling complexes, promote cooperative stimulus control of their associated signaling kinases. Here, we used structural lesions at the communication interface between core complexes to create an *Escherichia coli* strain with functional but dispersed signaling complexes. This strain allowed us to directly study how networking of signaling complexes affects chemotactic signaling and gradient-tracking performance. We demonstrate that networking of receptor complexes provides bacterial cells with about 10-fold-heightened detection sensitivity to attractants while maintaining a wide dynamic range over which receptor adaptational modifications can tune response sensitivity. These advantages proved especially critical for chemotaxis toward an attractant source under conditions in which bacteria are unable to alter the attractant gradient.

## IMPORTANCE

Chemoreceptor arrays are found in many motile bacteria. However, although our understanding of bacterial chemotaxis is quite detailed, the signaling and behavioral advantages of networked receptor arrays had not been directly studied in cells. We have recently shown that lesions in a key interface of the *E. coli* receptor array diminish physical connections and functional coupling between core signaling complexes while maintaining their basic signaling capacity. In this study, we exploited an interface 2 mutant to show, for the first time, that coupling between core complexes substantially enhances stimulus detection and chemotaxis performance.

## INTRODUCTION

Motile bacteria use a dedicated chemosensory system to track gradients of chemicals in their environment, moving toward attractant sources, such as amino acids, and away from potentially harmful repellents. In the bacterium *Escherichia coli*, five types of transmembrane receptors sense environmental signals and control the activity of an associated cytoplasmic histidine autokinase, CheA (1–3). CheA controls the cell’s swimming behavior by donating phosphoryl groups to the response regulator CheY. Phospho-CheY in turn binds to the base(s) of the flagellar motor(s), enhancing the probability of clockwise (CW) rotation, which causes random direction changes. Counterclockwise (CCW) motor rotation, the default behavior, produces forward swimming episodes (termed “runs”). A dedicated phosphatase, CheZ, counters CheA activity, ensuring rapid changes in intracellular phospho-CheY levels following receptor-promoted modulation of CheA activity.

*E. coli* senses spatial chemical gradients by comparing chemoeffector levels at a given time point to those experienced over the previous few seconds. This temporal sensing strategy effectively enables the cell to assess chemoeffector concentration changes over its forward-swimming run length. Such concentration comparisons are made possible by a sensory adaptation system that creates a memory of the recent chemical past in the form of covalent modifications to the receptor molecules. Receptor methylation, mediated by the enzyme CheR, and receptor deamidation and demethylation, mediated by the enzyme CheB, counter the effect of ligand on the receptor activity state. However, the sensory adaptation enzymes operate more slowly than do ligand-induced changes in receptor signal output, producing a several-second lag in adaptational updates and, thus, in “memory” (4–6).

The chemoreceptors in many bacterial species form two-dimensional arrays composed of thousands of signaling proteins (7). In *E. coli*, receptor arrays are built from core signaling units, which comprise two trimers of receptor homodimers linked by one CheA homodimer and two CheW adaptor proteins (8). Networking connections between these core signaling units form large arrays (7, 9–12). In the absence of CheA and CheW, the direct response of receptor trimers to ligand binding is not cooperative (13, 14). However, the kinase responses in networked receptor arrays can be highly cooperative (15–17), indicating that their activity controls are effectively coupled. Although the molecular basis for this coupling is not yet clear, various phenomenological models, based on the Monod-Wyman-Changeaux (MWC) model of allosteric transitions in proteins (18), have been successfully applied to quantitatively describe the signaling properties of this system (3, 16, 19, 20) and the way in which it controls bacterial behaviors (21). However, to date there have been no direct comparisons of the signaling and behavioral properties of cells with uncoupled core-signaling complexes versus cells with networked signaling arrays.

Cryo-EM and crystallography studies refined the structure of the *E. coli* chemosensory array and led to proposals that an interaction between CheW and the P5 domain of CheA (interface 2) was the key structural link between core signaling units in the array (9, 12, 22). We recently showed that amino acid replacements in CheW or CheA at predicted interface 2 residues both weakened the connections between receptor signaling units and dispersed the arrays (23). In cells lacking the sensory adaptation enzymes, interface 2 lesions did not alter the level of kinase activity or the ligand-dependent regulation of the kinase by the receptors, but they essentially abolished the cooperativity of the kinase activity responses. These results imply greatly reduced coupling between receptor signaling units and mirror previous *in vitro* measurements of receptor signaling complexes embedded in nanodiscs (24).

In the present work, we studied the behavior of a prototypical interface 2 defect in adaptation-proficient cells to directly assess the contribution of receptor networking to stimulus signaling and gradient-tracking performance. We found that in a high-activity state, dispersed receptor complexes were more sensitive and less cooperative than their networked counterparts. However, in a low-activity state, such as is typical of adaptation-proficient cells, dispersed receptor complexes were less sensitive than their networked counterparts. Indeed, the kinase activity in adaptation-proficient cells with native receptor arrays was approximately 10-fold more sensitive to attractant stimuli than it was in cells with dispersed signaling units. We further show that adaptation of receptors in dispersed signaling complexes is slower than in extended arrays but can be enhanced by overexpression of the adaptation enzymes. Extended receptor arrays proved advantageous for chemotaxis in attractant gradients and especially critical for chemotaxis toward a local and nonmetabolizable attractant source, which mimics conditions under which bacteria are too sparse to significantly affect attractant distribution. Analysis of the attractant distribution under those conditions suggested that the sensitivity gained through networked receptor arrays is indeed critical for sensing such chemoeffector gradients.

## RESULTS

### Interplay of receptor activity and core unit networking.

**FIG 1:**
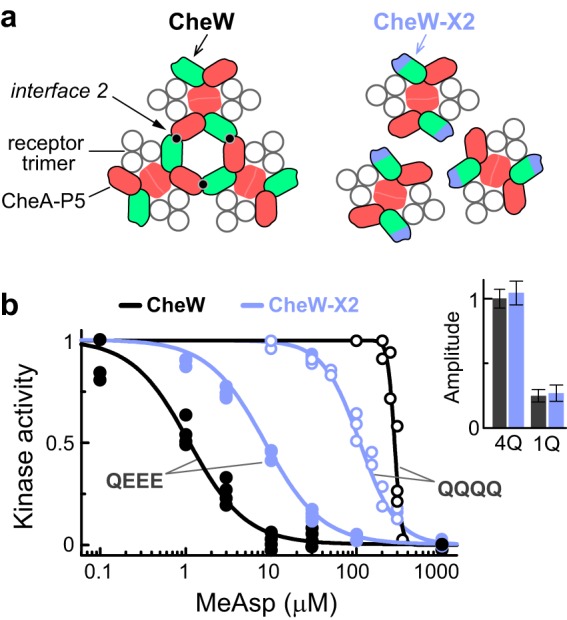
The effect of the CheW-X2 gene mutation on signaling in RP437-derived adaptation-deficient cells expressing Tar as the sole receptor. (a) Arrangement of core signaling complexes in wild-type or CheW-X2 cells. (b) Dose-response plots of normalized kinase activity in wild-type (black symbols) or CheW-X2 (blue symbols) strains expressing either Tar [QQQQ] receptors (open symbols) or Tar [QEEE] receptors (filled symbols) and the CheY-mCherry/CheZ*-mYFP FRET pair. Fits are to a multisite Hill function. Hill coefficients were 10 (CheW^+^, Tar [QQQQ]), 1.8 (CheW-X2, Tar [QQQQ]), 1.3 (CheW^+^, Tar [QEEE]), and 1.3 (CheW-X2, Tar [QEEE]). The magnitudes of the kinase activity responses to 1 mM MeAsp are also shown (normalized to the 4Q wild-type response; inset). Measurements were done at 30°C.

Amino acid replacements at interface 2 residues in CheW subdomain 1 or in CheA-P5 subdomain 2 weaken the associations between core signaling units (Fig. 1A) and thus disrupt receptor clustering and signaling cooperativity in adaptation-deficient cells (23). For the present study, we chose interface 2 lesions in CheW because they not only impair the CheW-P5 array interaction but also should affect an analogous CheW-CheW interaction (9), which also occurs in receptor arrays (25). A doubly mutant CheW (designated CheW-X2) with amino acid changes at two residues, R117D and F122S, exhibited properties comparable to those of previously characterized interface 2 mutant proteins: reduced efficiency of cross-linking to CheA-P5 (see Fig. S1A and B in the supplemental material), greatly impaired receptor array formation (Fig. S1C), reduced homo-fluorescence resonance energy transfer (homo-FRET) interactions between monomeric yellow fluorescent protein (mYFP)-tagged receptor molecules (Fig. S1D), and absence of the slow homo-FRET response to an attractant stimulus that occurs in native arrays (26).

We first characterized the signaling consequences of the CheW-X2 gene mutations with *in vivo* FRET kinase assays (27) in a strain lacking all chemoreceptors as well as the CheR and CheB adaptation enzymes. In such cells, plasmid-encoded receptor molecules have uniformly unmodified adaptation site residues: E residues represent unmethylated sites, whereas Q residues impart signaling properties similar to those produced by methylated E residues (28–30). An aspartate receptor with Q residues at all four protomer modification sites, Tar [QQQQ], produced high kinase activity with both the wild-type and CheW-X2 proteins, and a low-methylation mimic receptor, Tar [QEEE], exhibited lower kinase activity with both CheW proteins (Fig. 1B, inset). With high-activity Tar [QQQQ] receptor complexes, the CheW-X2 gene mutations reduced the cooperativity of the kinase response to ligand and shifted the response *K*_1/2_ (the attractant concentration that produces 50% inhibition of the kinase activity) to a lower concentration (Fig. 1B, open symbols). In contrast, with low-activity Tar [QEEE] receptor complexes, the CheW-X2 gene mutations shifted the response *K*_1/2_ to a higher ligand concentration (Fig. 1B, filled symbols). Thus, the relative detection sensitivities of the wild-type and CheW-X2 strains depend on receptor activity state. This property of networked signaling units provides wild-type cells with a wider dynamic range over which receptor modifications can modulate their response sensitivity.

### Interplay of the sensory adaptation system and core unit networking.

**FIG 2:**
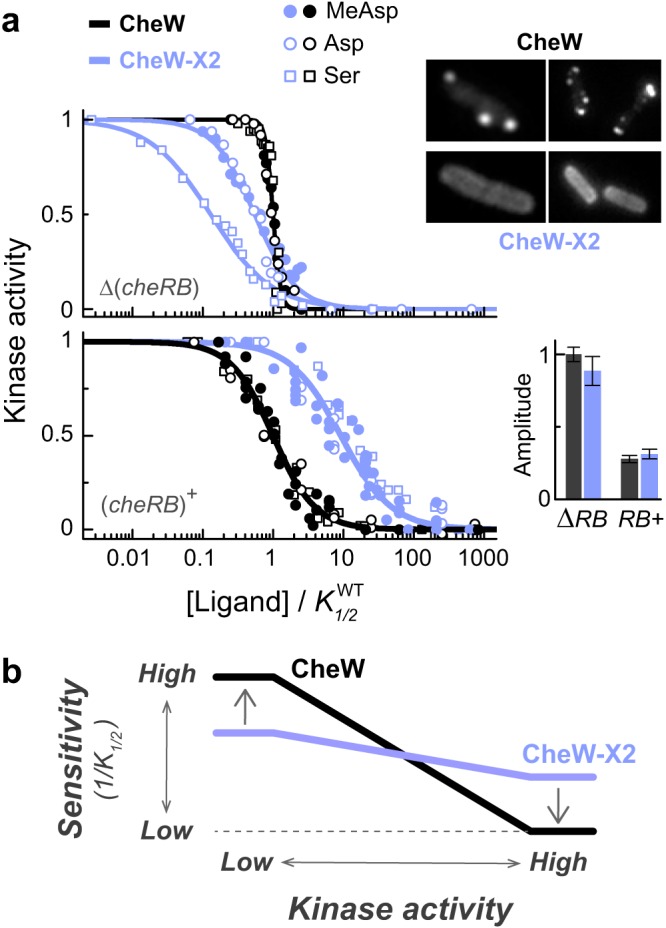
The effect of the CheW-X2 gene mutation on signaling in MG1655-derived cells. (a) Kinase activity dose responses measured in adaptation-deficient [Δ(*cheRB*)] or adaptation-proficient [(*cheRB*)^+^] cells with wild-type CheW (black lines and symbols) or CheW-X2 (blue lines and symbols). To facilitate comparisons between the responses to the different stimuli, ligand concentrations are normalized to the *K*_1/2_ value for that ligand in the wild-type CheW cells. The *K*_1/2_ values in Δ(*cheRB*) cells were 38 µM (serine), 40 µM (MeAsp), and 1.5 µM (Asp). The *K*_1/2_ values in (*cheRB*)^+^ cells were 0.23 μM (serine), 4.8 μM (MeAsp), and 0.4 μM (Asp). (Inset) Fluorescence images of wild-type or CheW-X2 cells expressing CheA::mYFP (left images) or Tar-mYFP (right images). The magnitudes of the saturated kinase activity responses [normalized to the Δ(*cheRB*) wild-type response] are also shown. (b) Schematic summary of the relationship between kinase activity and ligand sensitivity of signaling complexes in wild-type and CheW-X2 cells. The qualitative behavior depicted here was seen in both adaptation-deficient [Δ(*cheRB*)] and adaptation-proficient [(*cheRB*)^+^] backgrounds.

To explore the impact of networked receptor arrays on signaling and behavior in cells with a native chemotaxis system, we introduced the CheW-X2 gene mutations into the chromosome of MG1655 (IS*1*), a strain with robust chemotactic behavior (31–33). As expected, the CheW-X2 mutant derivative expressed the mutant CheW protein at the wild-type level (Fig. S2) but abrogated receptor clustering, as reported by fluorescence microscopy with tagged receptors (Tar-mYFP), tagged kinase (CheA::mYFP), or tagged CheR (mYFP-CheR). All reporter proteins formed high-contrast clusters in the wild-type strain but showed more uniform fluorescence distributions over the cell membrane of its CheW-X2 mutant derivative (Fig. 2A inset and Fig. S3A to C).

To monitor the kinase activity responses in MG1655 and its CheW-X2 gene mutant, we made derivatives of these strains deleted for the *cheY* and *cheZ* genes and introduced a plasmid encoding CheY and CheZ proteins fused to FRET donor and acceptor fluorophores. We also made versions of the two strains deleted for the *cheR* and *cheB* genes as well. We tested ligand responses to serine, an attractant detected by the Tsr receptor, and to aspartate or alpha-methyl-aspartate (MeAsp), attractants detected by the Tar receptor. The relative amplitudes of the kinase responses to the different stimuli are shown in Fig. S4. In the absence of the adaptation enzymes, the CheW-X2 gene mutations led to responses that were more sensitive (and less cooperative) than those of the wild type (Fig. 2A, upper panel). (The *K*_1/2_ shift was larger for the serine response than for the aspartate response, presumably because Tsr and Tar have intrinsically different signaling properties, such as activity biases.) In contrast, in the adaptation-proficient background, where the kinase activity is lower (Fig. 2A, inset), the *K*_1/2_ value for the CheW-X2 cells was higher than that for the wild-type cells (Fig. 2A, lower panel). Thus, networked receptor arrays in adaptation-proficient cells confer approximately 10-fold-increased sensitivity of ligand detection (lower *K*_1/2_).

These experiments revealed that the effect of the CheW-X2 gene mutations on signaling depends on the kinase activity state of the receptor complexes (Fig. 2B). In cells with only Tar [QQQQ] (Fig. 1B, open symbols) or with the native receptor repertoire at the QEQE state (Fig. 2A, upper panel), the signaling complexes have high kinase activities, and interface 2 connections between them impede their response to ligand, leading to lower sensitivity (but high cooperativity). In contrast, in cells with only Tar [QEEE] (Fig. 1B, filled symbols) or in adaptation-proficient cells with the native receptor repertoire (Fig. 2A, lower panel), the signaling complexes have substantially lower kinase activities, and interface 2 connections between them enhance their sensitivity to ligand.

**FIG 3:**
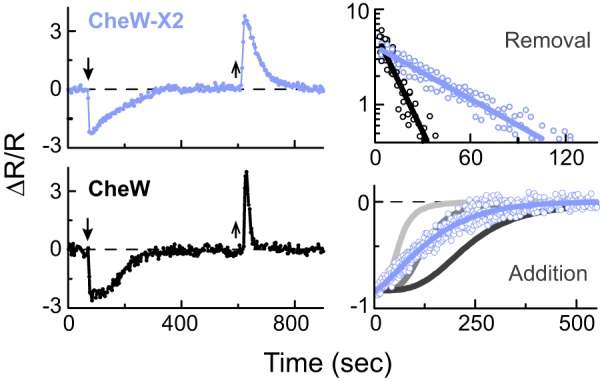
Adaptation kinetics of CheW-X2 and wild-type cells. (Left plots) Kinase activity over a prolonged period of exposure to a saturating MeAsp stimulus (between the arrows): 100 µM for wild-type cells (black lines and symbols) and 3,000 µM for CheW-X2 cells (blue lines and symbols). *R*, intensity ratio of the donor and acceptor emission channels. (Right plots) Adaptation time courses for the CheW-X2 cells (blue lines and symbols) following the removal or addition of 3,000 μM MeAsp. MeAsp concentrations for the wild-type cells were 30 μM (light gray line), 100 μM (dark gray line), and 3,000 μM (black line and symbols). For clarity, data symbols were omitted for the wild-type-addition experiments.

Despite different detection sensitivities, both the adaptation-proficient wild-type and CheW-X2 strains regained their prestimulus level of kinase activity following exposure to a saturating attractant stimulus (Fig. 3). However, the recovery time course in wild-type cells had an apparent initial lag which was previously attributed to cooperative methylation state control of receptor activity (34–36). This lag period is clearly missing in the CheW-X2 strain, consistent with the reduced cooperativity observed in the response of these cells to ligand (Fig. 2). Moreover, upon subsequent attractant removal, the wild-type cells exhibited a faster return to their prestimulus kinase activity than did CheW-X2 cells (Fig. 3). The low recovery rate of the CheW-X2 cells was largely alleviated by overexpression of the CheR and CheB adaptation enzymes from an inducible plasmid (Fig. S5). Evidently, CheB, which is primarily involved in adaptation to attractant decreases, is more rate-limiting in the CheW-X2 cells than is CheR, which is primarily involved in adaptation to attractant increases.

### The effect of networked receptor arrays on chemotaxis performance.

**FIG 4:**
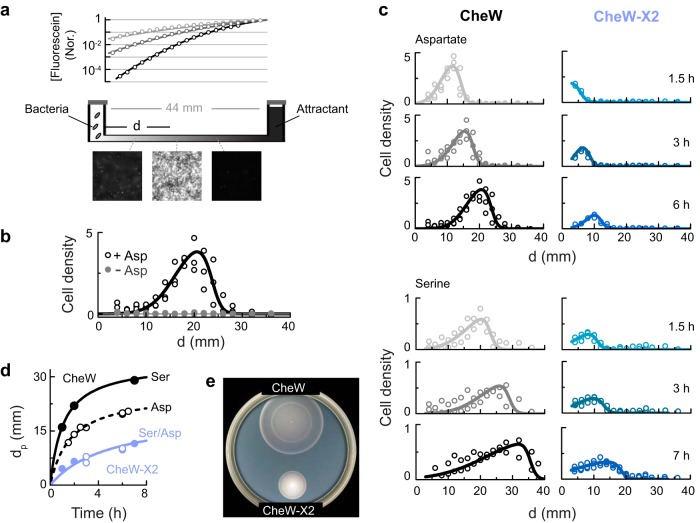
Chemotaxis performance in diffusion-generated attractant gradients. (a) Schematic depiction of the channel chemotaxis assay and fluorescein distribution along the channel (normalized [Nor.] to its density at the right chamber) 24 h (black), 50 h (dark gray), and 100 h (gray) after it was introduced to the right-side chamber. Lines represent *C*(*d*) = *C*_0_ ⋅ {*1* − *erf*[*d*/(4 ⋅ *D* ⋅ *t*)^1/2^]}, with *D* = 0.54 ⋅ 10^−5^ cm^2^/s. Typical bacterial images along the channel are also shown. (b) The distribution of wild-type cells 6 h after their introduction into the left-side chamber in the absence of a gradient (gray symbols) or after establishing an aspartate gradient, with *C*_0_ = 5 mM and *t*_*D*_ = 48 h (black symbols). “Cell density” corresponds to OD(*d*)/OD_0_ (see Materials and Methods). (c) The distribution of the wild-type and CheW-X2 cells in the channel at various times after introducing cells to the left side of the channel. Aspartate (upper part) or serine (lower part) gradients were pre-established in the channels (5 mM/48 h) prior to the addition of cells. Experiments were done at 30°C. (d) The time dependence of the bacterial distribution peak distance (*d*_*p*_) for experiments similar to those whose results are shown in panel c. (e) Colonies of wild-type and CheW-X2 cells in tryptone soft-agar plates.

To test the effect of networked receptor arrays on the ability of bacteria to travel toward an attractant source, we developed a chemotaxis assay based on a thin (150-μm) but long (44-mm) channel that is permeable to oxygen (Fig. 4A). The channel was initially filled with a motility buffer optimized for chemotaxis under conditions that do not support bacterial growth (37). Then, attractant was applied (at concentration *C*_0_) to one side of the channel and allowed to diffuse in the channel for time *t*_*D*_. For a channel of this length, a diffusion period of days is required to establish the attractant gradient (Fig. 4A, upper part). Given that this time is still much too short to allow equilibration of the ligand concentration in the device and that the volume of the channel is only one-third that of one side of the chamber in the device, even after partial diffusion into the channel, the attractant concentration within the side chamber remains near its initial value. After establishing an attractant gradient in the channel, at a time defined as *t* = 0, a low-density bacterial suspension (optical density at 600 nm [OD_600_] of 0.1) was added to the chamber at the downgradient end of the channel and the cell distribution along the channel was followed over time.

In the absence of attractant, wild-type cells did not travel along the channel even after 6 h (Fig. 4B; gray symbols). This was expected based on the estimated effective diffusion constant of randomly swimming cells (<10^−5^ cm^*2*^/s) (1). However, wild-type cells clearly accumulated in the channel in response to an aspartate or serine gradient (Fig. 4B; black symbols). Cell accumulation was apparent at 1.5 h, and movement toward the attractant source continued thereafter (Fig. 4C). Aspartate and serine gradients elicited qualitatively similar behaviors (Fig. 4C); however, cells traveled faster in the serine gradient and their density profile was less symmetric. These differences might reflect differences in the metabolism of the two attractants. The CheW-X2 and wild-type cells had similar kinase activity levels (Fig. 1 and 2), similar motor switching statistics (Fig. S6A), and similar overall CW bias distributions (Fig. S6B). However, the CheW-X2 cells progressed up the gradient less efficiently, in both cell numbers and rate of travel (Fig. 4C and D). The rate difference was qualitatively consistent with an approximately 50% slower expansion of the mutant colonies in tryptone soft-agar plates (Fig. 4E).

**FIG 5:**
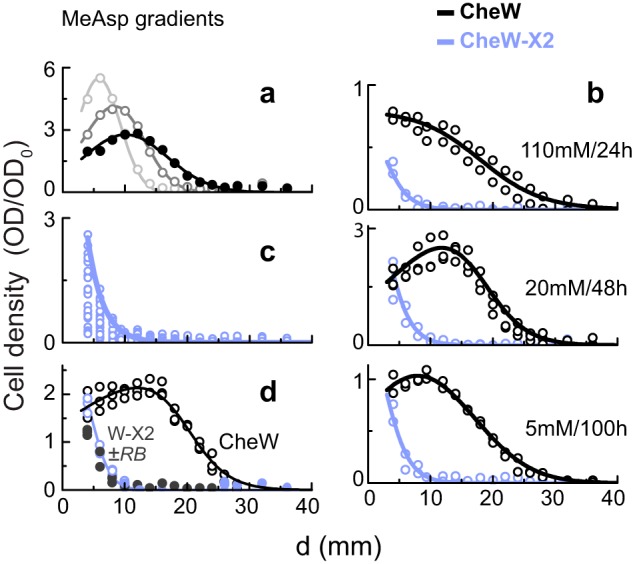
Chemotaxis performance in MeAsp gradients. (a) The distribution of wild-type cells along the channel with an established MeAsp gradient (*C*_0_ = 5 mM/*t*_*D*_ = 48 h) at 1.5 h (light gray), 3 h (dark gray), and 6 h (black) after cell addition. (b) The distribution of wild-type (black lines and symbols) or CheW-X2 (blue lines and symbols) cells at 6 h after their addition to the device with various MeAsp gradient parameters, *C*_0_ and *t*_*D*_, as indicated. (c) The distribution of CheW-X2 cells along the channel 6 h after adding the cells to the left-side chamber in various MeAsp gradients. See Fig. S7 for the full list of conditions and the corresponding expected gradients. The line, proportional to Exp(*−d*/2.5), describes the general bacterial distribution found here. (d) Bacterial distributions 6 h after introduction into a MeAsp gradient (*C*_0_ = 5 mM/*t*_*D*_ = 48 h). Wild-type cells are indicated with open black symbols, and CheW-X2 cells carrying an arabinose-inducible *cheRB* expression plasmid (pAV101) grown with no arabinose or with 0.007% arabinose are indicated with open blue symbols or filled gray symbols, respectively.

In serine or aspartate gradients, bacterial metabolism of the attractant can alter the local gradient profile. To test the capacity of cells to track a nonmetabolizable attractant gradient, we used the aspartate analog MeAsp. Moreover, because cell movements were followed for only a few hours, which is much less than the diffusion period (*t*_*D*_), the cells encountered a nearly constant MeAsp gradient. Wild-type cells moved up a MeAsp gradient (Fig. 5A and B, black symbols), whereas CheW-X2 cells hardly did (Fig. 5B, blue symbols). We tested a variety of gradients by adjusting the attractant concentration (*C*_0_) and the time allowed for diffusion into the channel prior to the addition of the cells (*t*_*D*_). The expected gradient profiles are shown in Fig. S7. Wild-type cells effectively tracked all gradients tested; however, the CheW-X2 cells failed to track any of the gradients and generally remained near the channel entrance (Fig. 5C). Overexpression of the sensory adaptation enzymes, which expedited adaptation of the CheW-X2 cells to attractant addition or removal (Fig. S5), did not improve their performance in the MeAsp gradient (Fig. 5D).

### A challenge in detecting a local attractant source.

**FIG 6:**
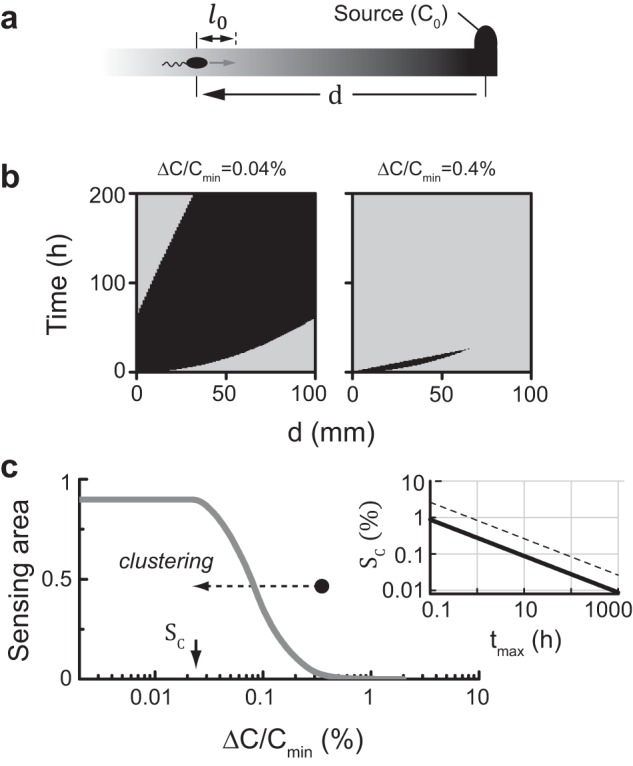
Factors affecting chemotaxis toward a local attractant source. (a) A schematic depiction of the problem: a local attractant source maintains a constant concentration *C*_0_ at the origin, and a cell is positioned at a distance *d* from the source. (b) Using *D* = 0.9 ⋅ 10^−5^ cm^2^/s, the attractant concentration *C*(*d*,*t*) and the relative change in attractant concentration (over *ℓ*_0_ = *~*10 μM) *ΔC*/*C*(*d*,*t*) were evaluated for various distances *d* and times *t* after the introduction of the source. Each point in the plots is marked in dark gray where both *C* > *C*_min_ and *ΔC*/*C* > *ΔC*/*C*_min_ at this position or in light gray where at least one of these conditions was violated. We assumed *C*_min_/*C*_0_ = 5 ⋅ 10^−7^, and the procedure was repeated for a *ΔC*/*C*_min_ value of 0.04% (left) or 0.4% (right). (c) The “sensing area”—the fraction of the time-distance space where both conditions are met—is plotted as a function of *ΔC*/*C*_min_. The critical gradient-detection sensitivity, *S*_*C*_, marks the point above which the “sensing area” begins to shrink. (Inset) The dependence of *S*_*C*_ on the maximal practical time, *t*_max_, allowed between the introduction of the source and its first detection by the cell for two values of *ℓ*_0_: 10 μm (solid line) and 30 μm (dashed line) (see also Text S1).

Given the inability of CheW-X2 cells to track the wide range of MeAsp gradients tested here (Fig. 5), we analyzed the behavioral challenges that such gradients may present to the cells. The chemotactic response of a bacterial cell at a distance *d* from a local attractant source (*C*_0_) (Fig. 6A) depends on its ability to detect a meaningful concentration change over its run length *ℓ*_0_ (10 to 30 μm for *E. coli*) (1). The efficiency of detection depends on the attractant concentration *C*(*d*,*t*) and its local gradient *ΔC*(*ℓ*_0_)/*C* at the location of the cell (38–40). However, in the case of a gradient emanating from a local source, these quantities are correlated. For the “constant source” discussed here, at any distance *d*, the attractant concentration increases monotonically with time while the local gradient monotonically decreases. Thus, shortly after introduction of the source, the attractant concentration at the cell’s location might be too low for the cell to effectively detect it, while at long times, the local gradient might be too shallow to elicit an effective chemotactic response. It follows that the tradeoff between local attractant concentration and gradient steepness limits the time window during which both factors are sufficiently large for an effective chemotactic response.

Is the time window during which the cell can respond to the gradient different for wild-type and CheW-X2 cells? If we assume for simplicity that the cells can efficiently respond to the gradient only if (i) *C*(*d*,*t*) *> C*_min_ and (ii) *ΔC*/*C*(*d*,*t*) *> ΔC*/*C*_min_, where *C*_min_ and *ΔC*/*C*_min_ represent detection properties of the cell, we can evaluate their ability to respond to the gradient at various times and distances from the source. In Fig. 6B, each point represents a certain time *t*_*D*_ after the introduction of the source and a certain distance *d* from the source. At each point, if both condition i and condition ii were met, the point was marked dark gray; if at least one of the conditions was violated, the point was marked light gray. This procedure was repeated for a relatively large value of *C*_0_/*C*_min_ (~10^7^) and two values of *ΔC*/*C*_min_, demonstrating that the capacity to detect the gradient can critically depend on *ΔC*/*C*_min_. This is further demonstrated in Fig. 6C, in which the area where both conditions are met (“sensing area”) is plotted as a function of *ΔC*/*C*_min_. The steep decline in the “sensing area” with increasing *ΔC*/*C*_min_ suggests that wild-type cells (when *ΔC*/*C*_min_ *< S*_*C*_) would respond to the gradient at almost any time (*t*_*D*_) or distance (*d*) from the source but that cells with 10-fold-higher *ΔC*/*C*_min_ would hardly respond to the gradient at any time or distance. The critical value *S*_*C*_, at which the “sensing area” starts to decline, can be evaluated by *ℓ*_0_/(*4 ⋅ D ⋅ t*_max_)^1/2^, and thus, it is slowly varying with *t*_max_, the maximal practical time allowed between the introduction of the source and its first detection by the cells (Fig. 6C, inset; see also Text S1 in the supplemental material). For *ℓ*_0_ = ~10 to 30 μm and *t*_max_ = ~10 to 100 h, *S*_*C*_ is about 0.1%, consistent with the observations made in reference 40 (assuming a run length of 10 μm). Thus, the time window during which cells can respond to the gradient depends critically on their basic gradient-detection capacity (*ΔC*/*C*_min_).

## DISCUSSION

Although chemoreceptor arrays have been found in many chemotactic bacteria (7), the signaling and behavioral advantages of networked receptors had not been experimentally demonstrated. In this study, we exploited an interface 2 array lesion that diminishes physical connections and functional coupling between receptor core signaling complexes to assess the contributions of clustered core complexes to stimulus detection and chemotaxis performance. In cells lacking the adaptation enzymes, we found that receptors in a high-activity state were less sensitive to an attractant ligand (had higher *K*_1/2_) in networked arrays (in wild-type cells) than as dispersed signaling complexes (in CheW-X2 cells). In contrast, in low-activity states, networked receptors were more sensitive than dispersed signaling complexes (Fig. 2B). We observed a similar tradeoff between kinase activity and detection sensitivity in adaptation-proficient cells: those with functionally coupled signaling teams were approximately 10-fold more sensitive to attractant stimuli than were cells with an interface 2 array defect. The idea that coupling between receptors can heighten sensitivity was explicitly suggested by Duke and Bray (41, 42). Further theoretical analysis of this system (20), based on the MWC model (16, 19), suggested that coupling between receptors can lead to enhanced sensitivity only when the receptors are in a low-activity state, while leading to high cooperativity when the receptors are in a highly active state. It was further suggested that if the coupling strength between receptors depends upon their signaling state, the *K*_1/2_ of the response can also shift when the receptors are in a high-activity state (43). These predictions were confirmed for cells with Tar receptors in mutationally imposed activity states (Fig. 1) and were consistent with the behaviors of cells under fully native conditions (Fig. 2).

The CheW-X2 array defect also altered the rate of sensory adaptation (Fig. 3), a likely manifestation of impaired networking connections between receptor core complexes. Cooperativity between signaling units is expected to alter the dependence of kinase activity on receptor methylation state and therefore should affect adaptation kinetics. In addition, extended receptor arrays can enhance the efficiency of the modification reactions, likely contributing to adaptation rate. Elevated expression of the CheR and CheB enzymes in the CheW-X2 background expedited their adaptation rate, but the profile of the adaptation time course was not identical to that seen with the wild type (Fig. S5). Receptor arrays can affect the efficiency of receptor modification in several ways. For example, receptor molecules are known to share access to CheR and CheB through “assistance neighborhoods” (44, 45). The level of this helping effect should be negligible between dispersed signaling units. In addition, CheB molecules activated by CheA-dependent phosphorylation have short half-lives and probably could not act efficiently on receptors in other core complexes unless they were structurally coupled through interface 2 connections.

To gauge the signaling benefits of extended receptor arrays, we examined the ability of wild-type and CheW-X2 cells to track local attractant gradients. In experiments with a metabolizable attractant gradient (serine/aspartate) or in tryptone semisolid agar plates, CheW-X2 cells showed substantial chemotaxis ability (up to 50% of that seen with the wild type) (Fig. 4). However, under nongrowth conditions at low cell densities, the CheW-X2 cells failed to progress up a gradient of nonmetabolizable attractant (Fig. 5). Thus, receptor arrays are especially critical for chemotaxis toward a local source of attractant that the bacteria are unable to modify. Such conditions include situations in which the bacterial density is low and they are unable to alter the gradient, circumstances that might be quite common in nature.

Why are extended receptor arrays critical for chemotaxis toward a nonmetabolizable attractant? At low ligand concentrations (*C* ≪ *K*_1/2_), the cell’s kinase response to ligand approximates a Hill function with power close to 1, so the kinase response *dA/dC*, at low concentrations, is approximately proportional to 1/*K*_1/2_. Thus, the signaling response to small changes in ligand concentration is expected to be 10-fold larger for receptor arrays in wild-type cells than for dispersed signaling units in CheW-X2 cells, leading to enhanced gradient-detection capacity (lower Δ*C*/*C*_min_) of wild-type cells. Analysis of the attractant gradients emanating by diffusion from a localized source (Fig. 6) indicated that the inverse relationship between the local attractant concentration *C*(*d*,*t*) and the local gradient Δ*C*/*C*(*d*,*t*) could indeed limit the ability of cells to sense the gradient. Moreover, these correlations can define a critical gradient-detection sensitivity *S*_*C*_, such that cells with Δ*C*/*C*_min_ > *S*_*C*_ cannot sense such gradients, independently of the source strength or time elapsed since its introduction (Fig. 6C). Taking the data together, this analysis suggests that the heightened sensitivity of wild-type cells, gained through networking of signaling complexes, is indeed critical for chemotaxis in such gradients. Detection sensitivity was less critical for performance at higher cell densities in metabolizable attractant gradients (Fig. 4E), conditions under which the local gradient is essentially independent of the local attractant concentration. Networking of core units might influence other signaling properties that contribute to the observed chemotaxis advantage of wild-type cells, for example, kinase activity fluctuations (46) or phospho-CheY distribution kinetics (47).

We suggest that the 10-fold increase in sensitivity gained by forming extended receptor arrays (Fig. 2) allows wild-type cells to overcome the sensing limits imposed by attractant diffusion (Fig. 6C, dashed arrow) and, together with the wider range of attractant concentrations to which they can respond (Fig. 1B), enables them to migrate effectively toward widely differing attractant sources.

## MATERIALS AND METHODS

### Bacterial strains and plasmids.

Strains and plasmids used in this work are listed in Table S1 in the supplemental material. The *E. coli* strains UU2567 (42), UU2806 (18), and VF7 are isogenic derivatives of parental strain RP437 (48). *E. coli* strains VF6, VF5, MK3, MK2, UU2942, and UU2943 are derivatives of the MG1655 (IS*1*) strain (33).

### Bacterial growth conditions.

Overnight cultures were diluted 100-fold in fresh tryptone broth (10 g/liter tryptone, 5 g/liter NaCl) supplemented with appropriate antibiotics and inducers and allowed to grow at 33.5°C with agitation to an OD_600_ of ~0.45. Cells were then washed twice in 10 ml motility buffer (10 mM potassium phosphate, 0.1 mM EDTA, 1 μM methionine, 10 mM lactic acid, pH 7.1). In the channel chemotaxis assay, 5 g/liter NaCl was also added to the buffer.

### Chemotaxis soft-agar assay.

Strains were assessed for chemotactic ability on tryptone soft-agar plates (10 g/liter tryptone, 5 g/liter NaCl, 2.5 g/liter agar) as previously described (49).

### Channel chemotaxis assay.

A long channel (length, 44 mm; height, 0.15 mm; width, 5 mm), permeable to oxygen (Ibidi μ-slide I^0.2^ Luer), was initially filled homogenously with motility buffer. Then, a solution containing the attractant to be tested was applied to one of the side chambers (~100 μl) embedded in 1% agarose gel. The channel was then sealed and incubated at 30°C for various periods to establish the gradient along the channel. Cells were grown as described above, except that here, to ensure high motility, cells were washed twice without an intermediate resuspension, followed by a gentle final resuspension in motility buffer to a final OD_600_ of 0.1. The cells expressed green fluorescent protein (GFP) from plasmid pSA11 (50), induced at 75 μM IPTG (isopropyl-β-d-thiogalactopyranoside). The low-density suspension was then applied to the opposite side of the channel, sealed, and incubated at 30°C. Several experiments were also done with denser cell suspensions diluted directly into the device, with no significant difference in the results. At various times after introduction of the cells, the channel was briefly mounted on an inverted Nikon Ti microscope (20×, 0.5 numerical aperture [NA]) equipped with a controlled *x*-*y* stage and an automatic focus system (set to the middle of the channel), and the GFP fluorescence was monitored along the channel (time required, ~2 min). In addition, a separate channel uniformly filled with the same cell suspension (OD_600_ = 0.1) was used to normalize the cell distribution. Experiments were performed in parallel with the wild-type and CheW-X2 strains using multiple channels for each condition.

### Receptor clustering tests.

Cells were grown as described above using the following inducer concentrations: 0.3 μM NaSal for CheA::mYFP/CheW; 13 μM IPTG for Tar-YFP; and 50 μM IPTG for mYFP-CheR. Cells were placed on a 1% agarose pad and covered with a coverslip. Fluorescence images were obtained at 30°C using a Nikon Ti inverted microscope equipped with a 100× Plan-Fluor objective (1.3 NA), a xenon lamp (Sutter Instruments), and a camera (Andor Technology). Images were then analyzed to extract clustering contrast values by computing the ratio of peak intensity (highest) to body intensity (mean of cell without the poles) in each cell after subtraction of the background fluorescence intensity.

### Fluorescence anisotropy measurement.

This technique has been described elsewhere (13, 51). In brief, cells were immobilized on a coverslip, placed in a flow chamber, and mounted on a Nikon fn1 microscope (32) at room temperature. The mYFP fluorophore was excited with linearly polarized light, and the emitted fluorescence was split using a polarizing beam splitter cube into parallel (*I*_par_) and perpendicular (*I*_per_) polarizations, which were monitored using two photon counters. The steady-state polarization of the emitted fluorescence is represented here by the fluorescence anisotropy *r*, defined as (*I*_par_ − *I*_per_)/(*I*_par_ + 2 ⋅ *I*_per_), where *I*_per_ has been corrected for imperfections in the optical system.

### *In vivo* FRET-based kinase assays.

The *in vivo* kinase assay measures CheA activity-dependent interactions between CheY and CheZ proteins tagged with donor and acceptor fluorophores (34, 27). Cell preparation and flow cell assembly were similar to those performed in the anisotropy assay described above. Two alternative pairs were used, with CheY and CheZ tagged with the mYFP/mCherry pair (32) or the mYFP/mCFP pair (38). The donors (mYFP and mCFP, respectively) were excited using unpolarized light, and fluorescence emission from the FRET donor and acceptor was continually monitored by the use of photon-counting photomultipliers. Dose-response curves were obtained by plotting the fractional changes in kinase activity versus the applied stimulus. Total CheA kinase activity was measured as the change elicited by a saturating stimulus or by 3 mM NaCN or KCN (52).

### Tethering assay.

Cells were grown and washed as described above. Cell suspensions were stirred for 8 s using a milk frother and washed twice in 10 ml of KEP buffer (10 mM KPO_4_, 0.1 mM K-EDTA; pH 7.0) and a third time in motility buffer. Cell suspensions (100 µl) were then mixed with 5 µl of anti-flagellin antibody (1:200 dilution) and placed on a KOH-treated coverslip for 30 min. Movies of the rotating cells (10 to 30 s each) were taken at 30°C at a frame rate of 160 Hz (Lumenera camera), using a 40× objective. Movies were analyzed using MatLab. The long axis of the cell was identified, and the change in the cell’s angle between frames was calculated. The direction of rotation was determined, taking into account the rotation speed and considering a minimal significant rotation to be larger than 7°.

### Cross-linking assays.

UU2806 cells cotransformed with pGP55 (23) (or pGP55 derivatives carrying CheW-X2 gene mutations) and pRR53 (53) or pPA90 (54) were treated with 300 μM Cu2+ for 10 min at 35°C to induce disulfide formation. Whole-cell lysates were separated by SDS/PAGE, and CheA-containing species were detected by Western blot analysis using a polyclonal anti-hemagglutinin (HA) antibody (Pierce).

## SUPPLEMENTAL MATERIAL

Figure S1  Characterization of the CheW-X2 protein in RP437 derivative strains. (A) *In vivo* detection of interface 2 cross-linking products. Cells of strain UU2806 [Δ(*cheA-cheZ*) Δ(*tar tap tsr trg aer*)] carried two compatible plasmids: (i) pRR53 (wild-type Tsr) or pPA90 (Tsr [290–551]) and (ii) a wild-type or CheW-X2 mutant derivative of pGP55 which coexpresses HA-tagged CheA-A546C and CheW-E27C, an array interface 2 cross-linking reporter pair (18). Cells were grown and treated as detailed in Materials and Methods, and lysate proteins were separated by SDS-PAGE and probed with anti-HA antibody to detect cross-linked CheA-CheW products. Experiments with wild-type Tsr were carried out at different expression levels (1× or 0.5× relative to chromosomally expressed Tsr in RP437). (B) Interface 2 cross-linking efficiency at different Tsr expression levels. The band profiles shown in panel A were quantified by densitometry, and the fraction of CheA cross-linked to CheW in each experiment was normalized to the cross-linking yield at each Tsr expression level for reporter proteins bearing no interface 2 lesions. Histogram bars show the means and standard errors of results from 3 to 5 independent experiments. (C) Clustering of core signaling units in wild-type and CheW-X2 cells. Strain UU1607 [Δ(*cheAW*)], which contains a wild-type complement of receptor proteins, carried plasmid pAV232 derivatives encoding CheA::mYFP together with either wild-type CheW or CheW-X2. Cells were imaged by fluorescence light microscopy. (D) Homo-FRET characterization of cells expressing mYFP-tagged receptors in wild-type or CheW-X2 cells. Strain UU2806 [Δ(*cheA-cheZ*) Δ(*tar tap tsr trg aer*)] carried two compatible plasmids: pAV45 to express the mYFP-tagged Tar [QQQQ] receptor and derivatives of pPM25 expressing CheA/CheW (black symbols) or CheA/CheW-X2 (blue symbols). Cells were illuminated with polarized light at the YFP excitation wavelength, and the extent of polarization (anisotropy) in the emitted light was measured (see Materials and Methods). MeAsp (1 mM) was added during the time indicated by the bar. Download Figure S1, PDF file, 1.1 MB

Figure S2  Protein levels of chromosomally expressed CheW-X2 in MG1655 (IS*1*). CheW protein levels were determined in strains VF6 (wild type) and VF5 (CheW-X2 gene mutant) transformed with pRR48 (as a source of β-lactamase) and grown at 30°C for 6 h with agitation (250 rpm) in tryptone broth containing 100 μg/ml ampicillin. Protein lysates were separated by SDS-PAGE and probed with polyclonal anti-CheW and anti-β-lactamase antibodies. Download Figure S2, PDF file, 1.4 MB

Figure S3  Clustering pattern of core signaling units in wild-type and CheW-X2 strains. Representative fluorescence images of wild-type and CheW-X2 mutant derivatives of strain MG1655 (IS*1*) expressing CheA::mYFP (A), Tar-mYFP (B), or mYFP-CheR (C). In each case, images of the two strains are shown using the same gray-level scale. Clustering contrast values were computed from the ratio of peak (highest) to body (mean of values measured for the cell without the poles) intensities in each cell. Distributions of clustering contrast scores are also shown. A total of 50 to 100 cells were analyzed for each experiment. Download Figure S3, PDF file, 2.1 MB

Figure S4  Kinase activity in Δ(*cheRB*) derivatives of MG1655 (IS*1*). Kinase activity was calculated from *in vivo* FRET-based kinase assays measuring the extent of FRET reduction elicited by exposing cells to a saturating dose of l-aspartate (1 mM) or of l-serine (1 mM) or of both attractants (1 mM each). Results are normalized to wild-type kinase activity upon treatment with l-aspartate and l-serine. Histogram bars show the means and standard errors (SE) of results from at least three independent experiments. Download Figure S4, PDF file, 1.4 MB

Figure S5  Adaptation kinetics of CheW-X2 cells overexpressing the CheR and CheB adaptation enzymes. (A) Kinase activity over the duration of a prolonged saturating MeAsp (3 mM) stimulus (red bar) for CheW-X2 cells with (red) or without (blue) overexpression of the adaptation enzymes CheR and CheB (plasmid pAV101 induced by 0.007% arabinose). R, relative intensities measured in the red and yellow channels. (B) Adaptation time course of the CheRB-overexpressing cells (red symbols) following the removal of MeAsp. The black and blue lines represent the corresponding dynamics in the wild-type and CheW-X2 cells, as described for Fig. 3. (C) Adaptation time course of the CheRB-overexpressing cells (red symbols) following the addition of MeAsp. The blue line represents the corresponding dynamics in the CheW-X2 cells, as described for Fig. 3. Download Figure S5, PDF file, 1.1 MB

Figure S6  Motor rotation properties in wild-type and CheW-X2 cells. Wild-type and CheW-X2 cells were tethered to glass slides, and their direction of rotation was analyzed as described in Materials and Methods. Presented are the normalized distribution of CW and CCW time intervals (A) and the histogram of CW bias—the fraction of time during which the cells rotated in the CW direction —(B) for wild-type (dark gray symbols) or CheW-X2 (blue symbols) cells. Download Figure S6, PDF file, 0.9 MB

Figure S7  The expected MeAsp distributions for the experiments whose results are shown in Fig. 5. The MeAsp distribution along the channel *C*(*d*) was estimated for the different combinations of *C*_0_ and *t*_*D*_, assuming a diffusion constant of *D* = 0.9 ⋅ 10^−5^ cm^2^/s and $C(d)/C_{0}=1-\text{erf}(d/\sqrt{4\;\cdot \;D\;\cdot \;t_{D}})$. Download Figure S7, PDF file, 0.9 MB

Table S1  Strains and plasmids. Table S1, PDF file, 0.2 MB

Text S1  Chemotaxis toward constant source, additional analysis. Download Text S1, PDF file, 0.2 MB
