# Supplementary figures and images for: Networked Chemoreceptors Benefit Bacterial Chemotaxis Performance
Source: mBio. 2016 Dec 20;7(6):e01824-16. doi: 10.1128/mBio.01824-16 (PMC5181776; doi:10.1128/mBio.01824-16)

**Figure S1.** Characterization of the CheW-X2 protein in RP437 derivative strains.

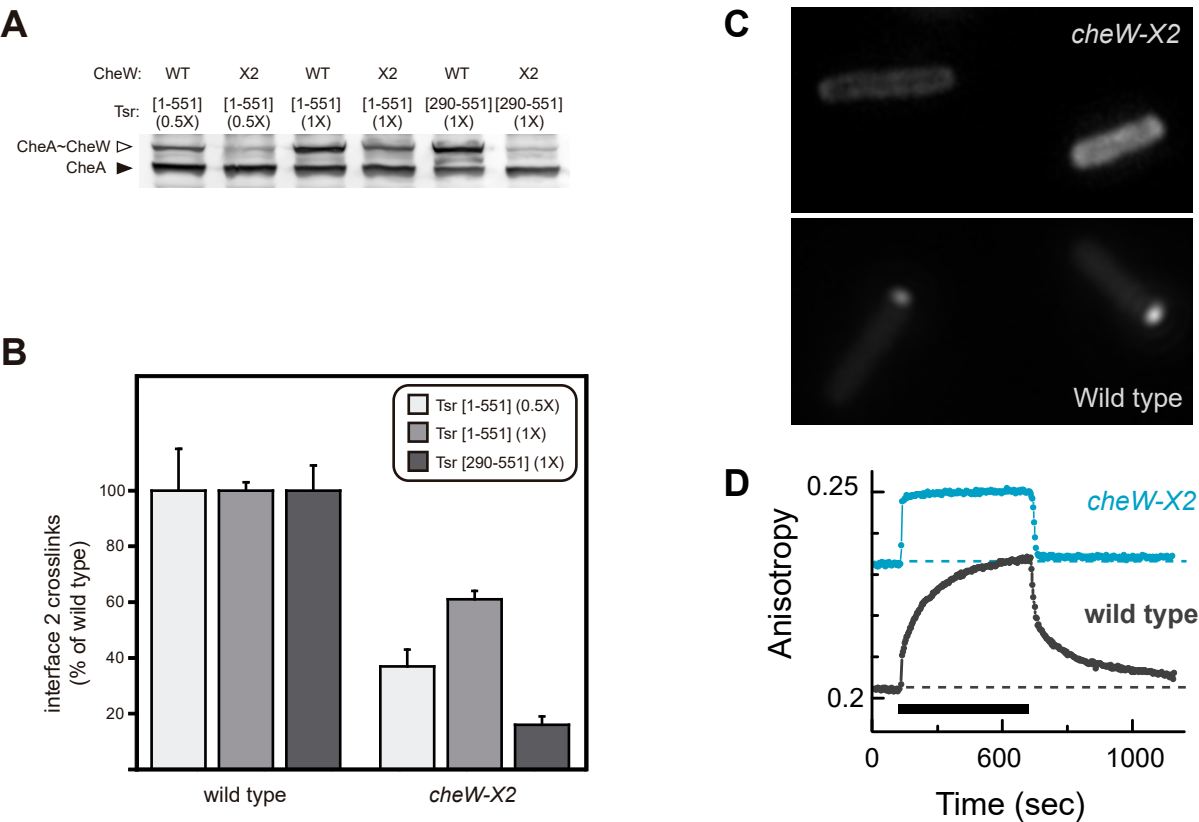

Supplement: Figure S1 — Characterization of the CheW-X2 protein in RP437 derivative strains. (A) In vivo detection of interface 2 cross-linking products. Cells of strain UU2806 [Δ(cheA-cheZ) Δ(tar tap tsr trg aer)] carried two compatible plasmids: (i) pRR53 (wild-type Tsr) or pPA90 (Tsr [290–551]) and (ii) a wild-type or CheW-X2 mutant derivative of pGP55 which coexpresses HA-tagged CheA-A546C and CheW-E27C, an array interface 2 cross-linking reporter pair (18). Cells were grown and treated as detailed in Materials and Methods, and lysate proteins were separated by SDS-PAGE and probed with anti-HA antibody to detect cross-linked CheA-CheW products. Experiments with wild-type Tsr were carried out at different expression levels (1× or 0.5× relative to chromosomally expressed Tsr in RP437). (B) Interface 2 cross-linking efficiency at different Tsr expression levels. The band profiles shown in panel A were quantified by densitometry, and the fraction of CheA cross-linked to CheW in each experiment was normalized to the cross-linking yield at each Tsr expression level for reporter proteins bearing no interface 2 lesions. Histogram bars show the means and standard errors of results from 3 to 5 independent experiments. (C) Clustering of core signaling units in wild-type and CheW-X2 cells. Strain UU1607 [Δ(cheAW)], which contains a wild-type complement of receptor proteins, carried plasmid pAV232 derivatives encoding CheA::mYFP together with either wild-type CheW or CheW-X2. Cells were imaged by fluorescence light microscopy. (D) Homo-FRET characterization of cells expressing mYFP-tagged receptors in wild-type or CheW-X2 cells. Strain UU2806 [Δ(cheA-cheZ) Δ(tar tap tsr trg aer)] carried two compatible plasmids: pAV45 to express the mYFP-tagged Tar [QQQQ] receptor and derivatives of pPM25 expressing CheA/CheW (black symbols) or CheA/CheW-X2 (blue symbols). Cells were illuminated with polarized light at the YFP excitation wavelength, and the extent of polarization (anisotropy) in the emitted light w [file mbo006163119sf1.pdf]

**Figure S4.** Kinase activity in  $\Delta(\text{cheRB})$  derivatives of MG1655 (IS1).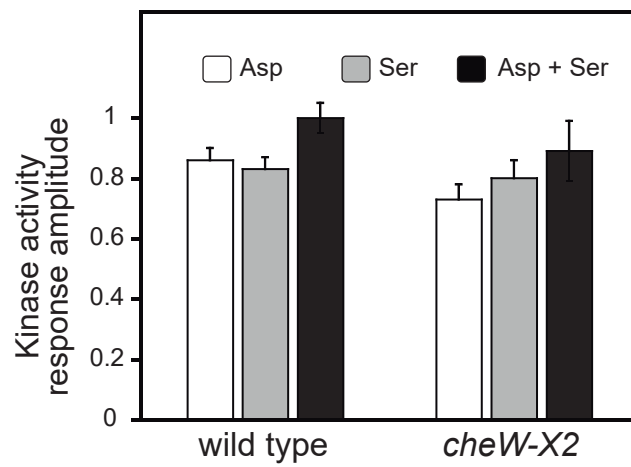

Supplement: Figure S4 — Kinase activity in Δ(cheRB) derivatives of MG1655 (IS1). Kinase activity was calculated from in vivo FRET-based kinase assays measuring the extent of FRET reduction elicited by exposing cells to a saturating dose of l-aspartate (1 mM) or of l-serine (1 mM) or of both attractants (1 mM each). Results are normalized to wild-type kinase activity upon treatment with l-aspartate and l-serine. Histogram bars show the means and standard errors (SE) of results from at least three independent experiments. Download [file mbo006163119sf4.pdf]

**Figure S5.** Adaptation kinetics of *cheW*-X2 cells over-expressing CheR and CheB.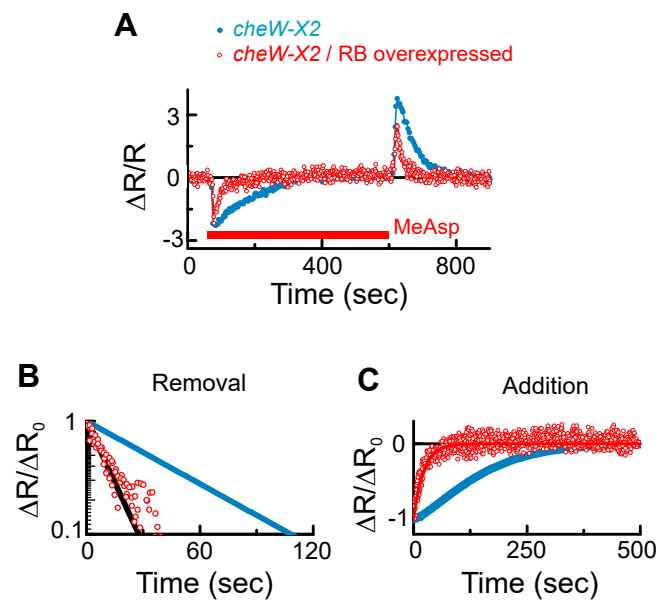

Supplement: Figure S5 — Adaptation kinetics of CheW-X2 cells overexpressing the CheR and CheB adaptation enzymes. (A) Kinase activity over the duration of a prolonged saturating MeAsp (3 mM) stimulus (red bar) for CheW-X2 cells with (red) or without (blue) overexpression of the adaptation enzymes CheR and CheB (plasmid pAV101 induced by 0.007% arabinose). R, relative intensities measured in the red and yellow channels. (B) Adaptation time course of the CheRB-overexpressing cells (red symbols) following the removal of MeAsp. The black and blue lines represent the corresponding dynamics in the wild-type and CheW-X2 cells, as described for Fig. 3. (C) Adaptation time course of the CheRB-overexpressing cells (red symbols) following the addition of MeAsp. The blue line represents the corresponding dynamics in the CheW-X2 cells, as described for Fig. 3. Download [file mbo006163119sf5.pdf]

**Figure S6.** Motor rotation properties in wild type and *cheW-X2* cells.

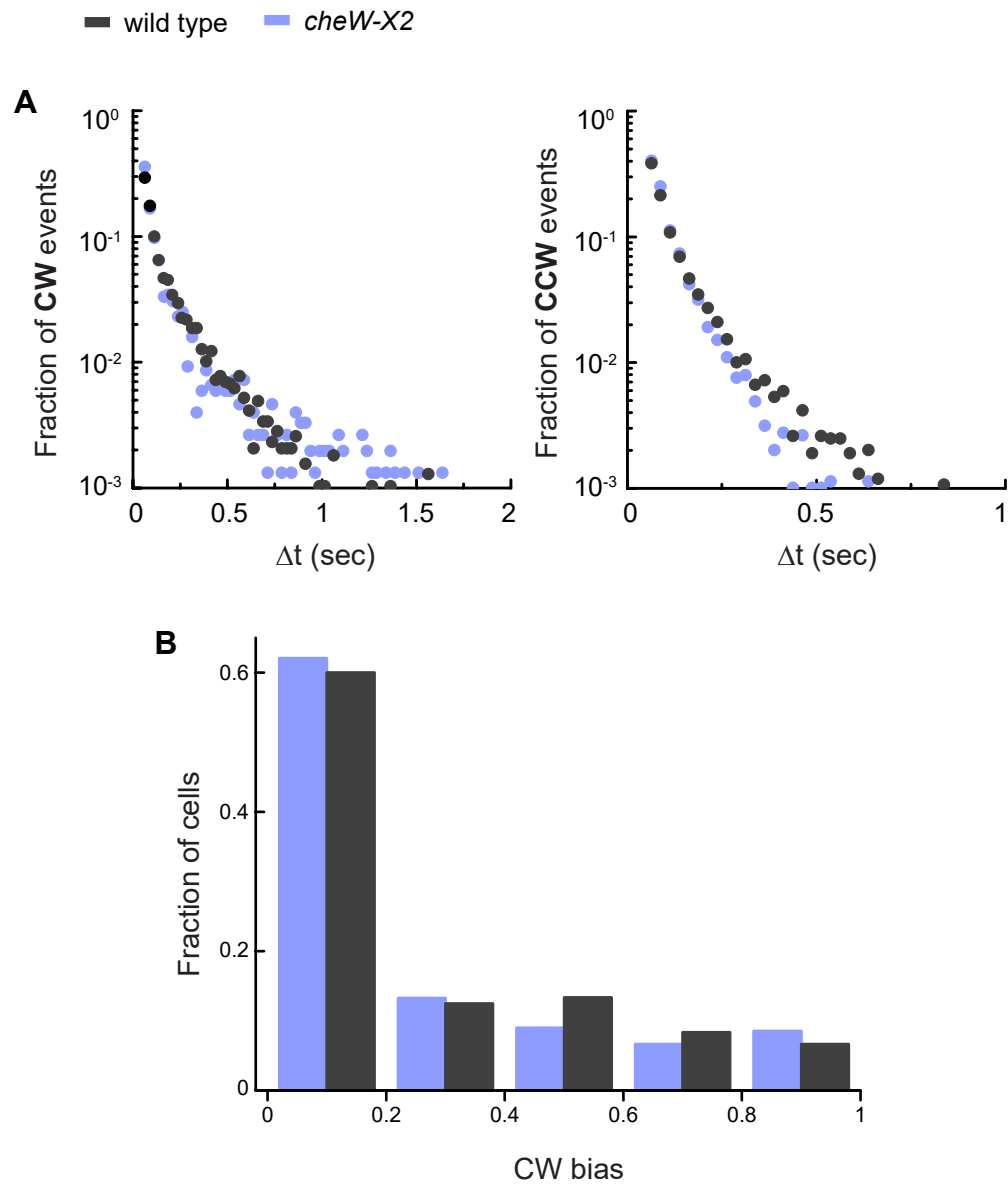

Supplement: Figure S6 — Motor rotation properties in wild-type and CheW-X2 cells. Wild-type and CheW-X2 cells were tethered to glass slides, and their direction of rotation was analyzed as described in Materials and Methods. Presented are the normalized distribution of CW and CCW time intervals (A) and the histogram of CW bias—the fraction of time during which the cells rotated in the CW direction —(B) for wild-type (dark gray symbols) or CheW-X2 (blue symbols) cells. Download [file mbo006163119sf6.pdf]

**Figure S7.** The expected MeAsp distributions for the experiments shown in Fig. 5.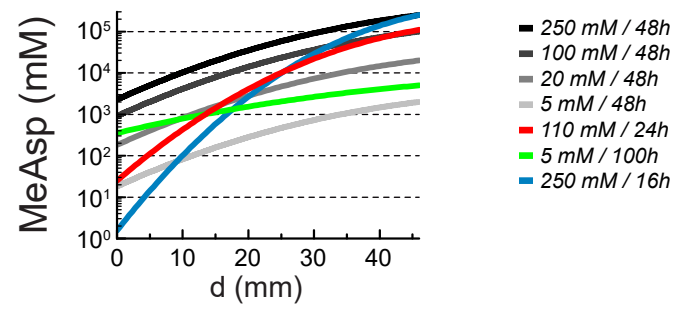

Supplement: Figure S7 — The expected MeAsp distributions for the experiments whose results are shown in Fig. 5. The MeAsp distribution along the channel C(d) was estimated for the different combinations of C0 and tD, assuming a diffusion constant of D = 0.9 ⋅ 10−5 cm2/s and C(d)/C0=1−erf(d/4 · D · tD). Download [file mbo006163119sf7.pdf]
