# Supplementary material for: Networked Chemoreceptors Benefit Bacterial Chemotaxis Performance
Source: mBio. 2016 Dec 20;7(6):e01824-16. doi: 10.1128/mBio.01824-16 (PMC5181776; doi:10.1128/mBio.01824-16)
Supplement: Figure S2 — Protein levels of chromosomally expressed CheW-X2 in MG1655 (IS1). CheW protein levels were determined in strains VF6 (wild type) and VF5 (CheW-X2 gene mutant) transformed with pRR48 (as a source of β-lactamase) and grown at 30°C for 6 h with agitation (250 rpm) in tryptone broth containing 100 μg/ml ampicillin. Protein lysates were separated by SDS-PAGE and probed with polyclonal anti-CheW and anti-β-lactamase antibodies. Download [file mbo006163119sf2.pdf]

Figure S2. Protein levels of chromosomally expressed CheW-X2 in MG1655 (IS1).

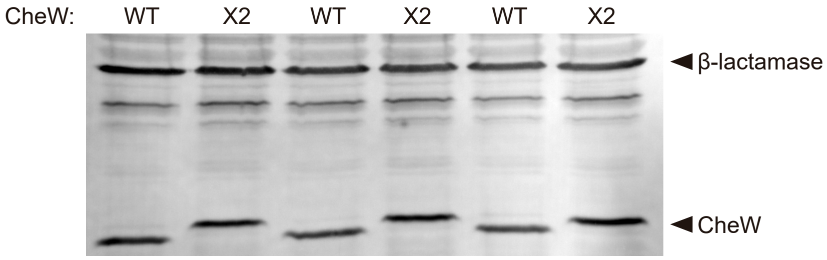

$$\frac{\text{CheW-X2 / }\beta\text{-lactamase}}{\text{CheW / }\beta\text{-lactamase}} \sim 1.16$$
