# Supplementary material for: Networked Chemoreceptors Benefit Bacterial Chemotaxis Performance
Source: mBio. 2016 Dec 20;7(6):e01824-16. doi: 10.1128/mBio.01824-16 (PMC5181776; doi:10.1128/mBio.01824-16)

Figure S3. Clustering pattern of core signaling units in wild type and *cheW-X2* strains.

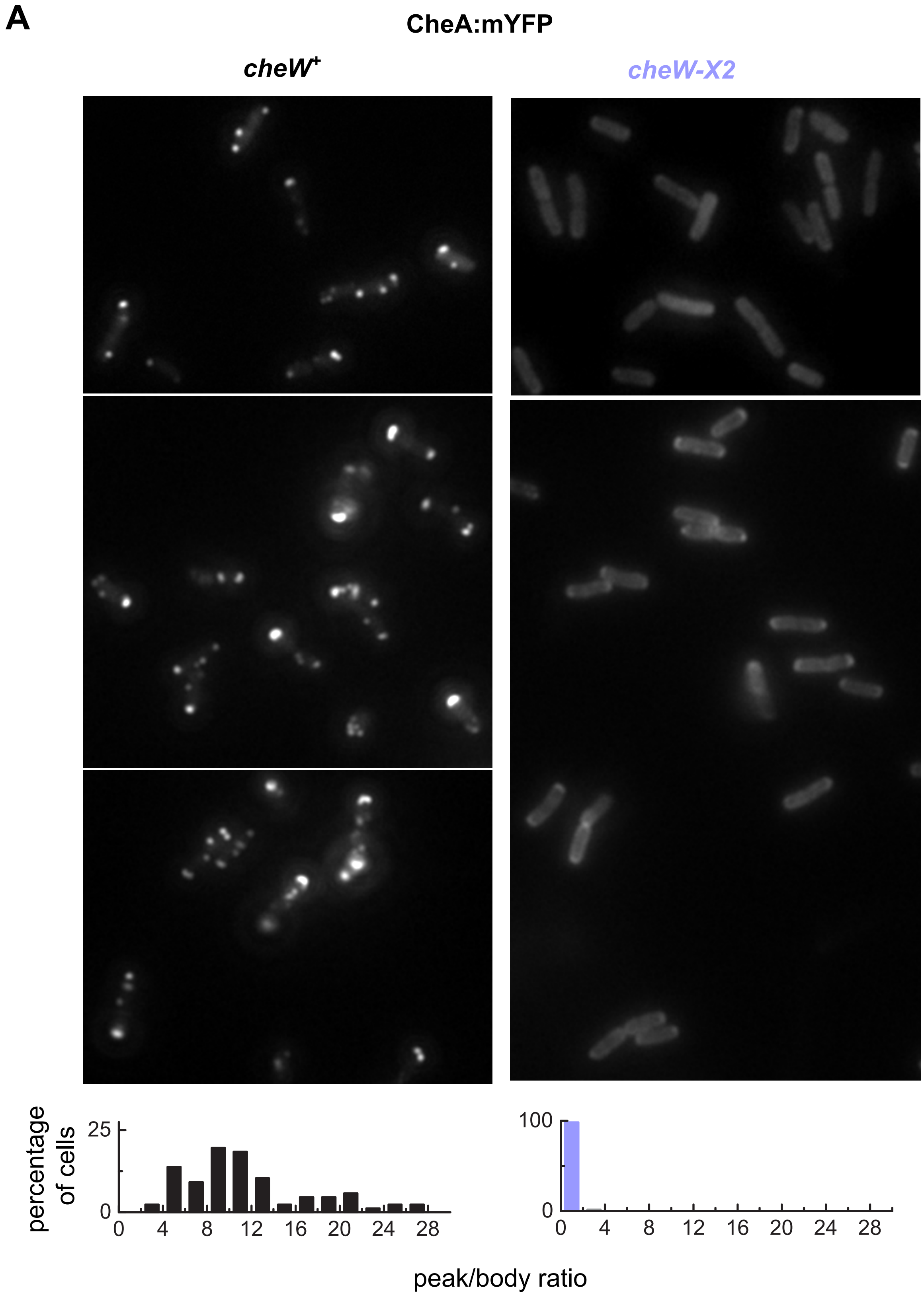

Figure S3. Clustering pattern of core signaling units in wild type and *cheW-X2* strains.

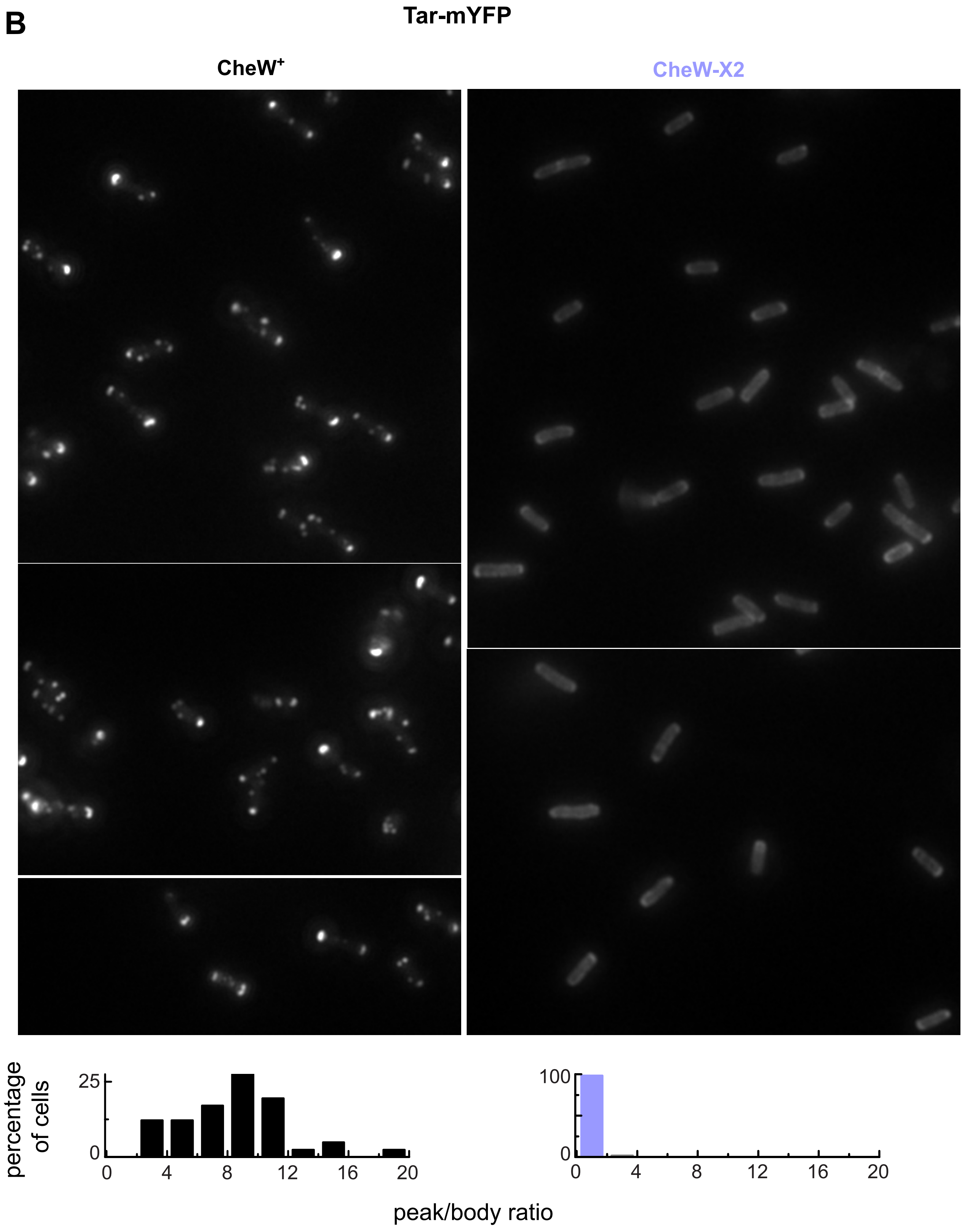

Figure S3. Clustering pattern of core signaling units in wild type and *cheW-X2* strains.

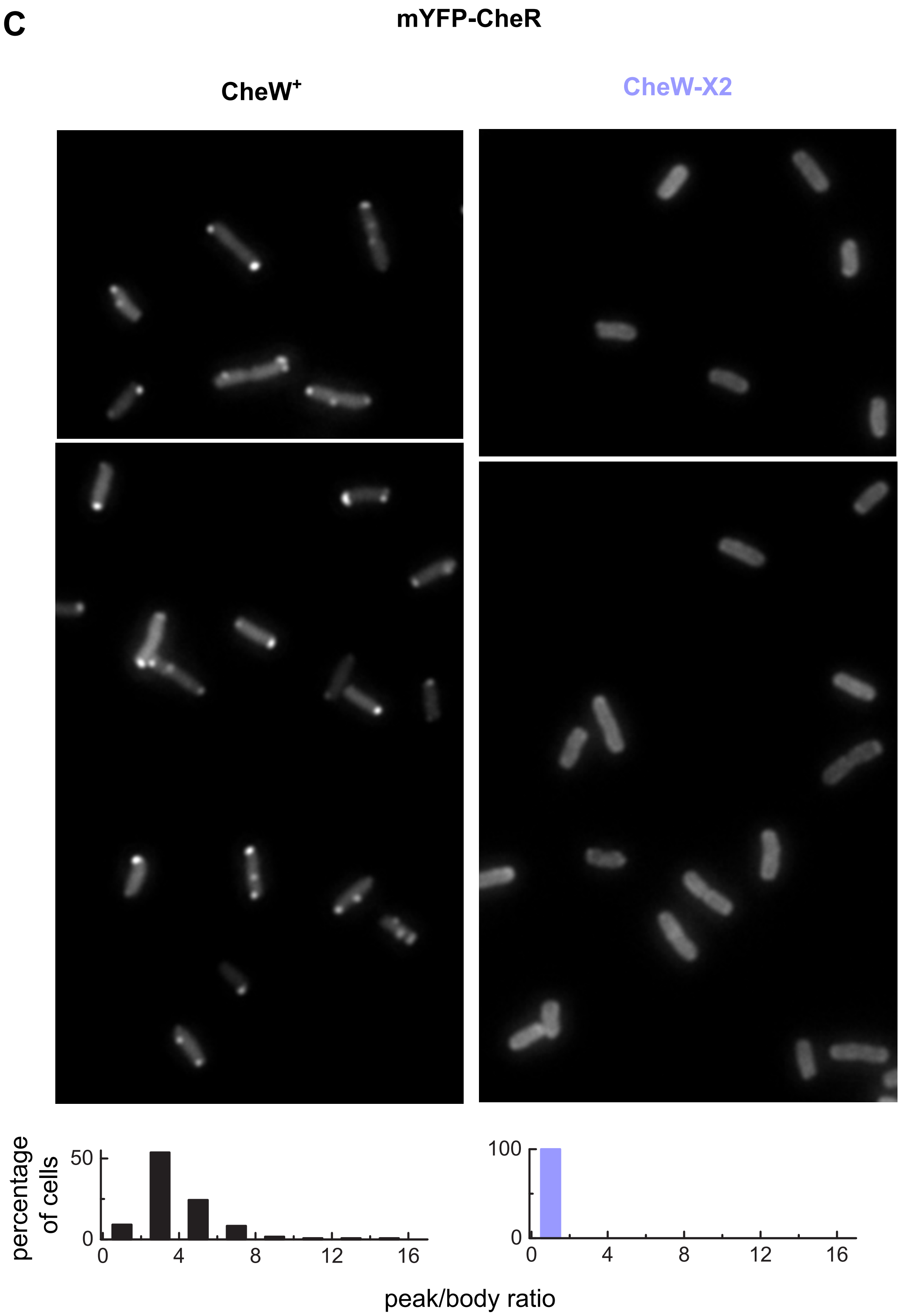

Supplement: Figure S3 — Clustering pattern of core signaling units in wild-type and CheW-X2 strains. Representative fluorescence images of wild-type and CheW-X2 mutant derivatives of strain MG1655 (IS1) expressing CheA::mYFP (A), Tar-mYFP (B), or mYFP-CheR (C). In each case, images of the two strains are shown using the same gray-level scale. Clustering contrast values were computed from the ratio of peak (highest) to body (mean of values measured for the cell without the poles) intensities in each cell. Distributions of clustering contrast scores are also shown. A total of 50 to 100 cells were analyzed for each experiment. Download [file mbo006163119sf3.pdf]
