# Supplementary material for: Networked Chemoreceptors Benefit Bacterial Chemotaxis Performance
Source: mBio. 2016 Dec 20;7(6):e01824-16. doi: 10.1128/mBio.01824-16 (PMC5181776; doi:10.1128/mBio.01824-16)
Supplement: Table S1 — Strains and plasmids. [file mbo006163119st1.pdf]

Table S1. Strains and plasmids.

strains	genotype	reference
**group 1**	derivatives of RP437	
UU2567	*Δ(tar-tap-cheR-cheB-cheY-cheZ)4211 Δtsr-5547 Δaer-1 Δtrg-4543*	(52)
VF7	*cheW-R117D/F122S* Δ(*tar-tap-cheR-cheB-cheY-cheZ*)*4211 Δtsr-5547 Δaer-1 Δtrg-4543*	this work
UU2806	Δ(*cheA-cheW-tar-tap-cheR-cheB-cheY-cheZ*)*1214* Δ*tsr*-5547 Δ*aer*-1 Δ*trg-4543*	this work
**group 2**	Derivatives of MG1655 (IS1)	this work
VF5	*cheW-R117D/F122S*	this work
VF6	wild type	this work
MK2	*cheW-R117D/F122S* Δ(*cheY-cheZ*)4211	this work
MK3	Δ(*cheY-cheZ*) 4211	this work
UU2942	Δ(*cheR-cheB-cheY-cheZ*)4211	this work
UU2943	*cheW-R117D/F122S* Δ(*cheR-cheB-cheY-cheZ*)4211	this work

plasmid	genotype of expression insert	reference
pAV44	*tar(Δ[528-563])-myfp*	(32)
pAV45	*tar([QQQQ] Δ[528-563])-myfp*	(32)
pAV76	*cheY-mCherry cheZ(F98S)-myfp*	(32)
pAV101	*cheR*$^{+}$ *cheB*$^{+}$	this work
pAV139	*tar [QQQQ]*	this work
pAV306	*tar [QEEE]*	this work
pAV232	*cheA(Δ[161-226]Ωmyfp) cheW*$^{+}$	(23)
pAV280	*cheA*$^{+}$ *cheW (R117D/F122S)*	this work
pAV287	*cheA(Δ[161-226]Ωmyfp) cheW(R117D/F122S)*	this work
pAV288	*myfp-cheR*	this work
pGP55	*cheA*(*M98L/C120S/C213S/C415S/A546C/S147ΩHA/A241 ΩHA*) *cheW*(E27C)	(23)

plasmid	genotype of expression insert	reference
pPA90	*tsr[290-551]*	(49)
pPM25	*cheA*$^{+}$ *cheW*$^{+}$	(23)
pRR48	*empty vector*	(48)
pRR53	*tsr[QEQE]*	(48)
pSA11	*gfp*	(50)
pVS88	*cheY-yfp cheZ-cfp*	(34)
