# Supplementary material for: Networked Chemoreceptors Benefit Bacterial Chemotaxis Performance
Source: mBio. 2016 Dec 20;7(6):e01824-16. doi: 10.1128/mBio.01824-16 (PMC5181776; doi:10.1128/mBio.01824-16)
Supplement: Text S1 — Chemotaxis toward constant source, additional analysis. Download [file mbo006163119s1.pdf]

Text S1. Chemotaxis towards constant source – addional analysis of Fig. 6

The expected attractant distribution $C(x)$ formed by a local and 'constant source' in a quasi 1D channel is:

$$C(x) = C_0 \cdot [1 - \mathrm{erf}(\eta)] \qquad \eta \equiv \frac{d}{\sqrt{4 \cdot D \cdot t}} \quad ; \quad \mathrm{erf}(\eta) \equiv \frac{2}{\sqrt{\pi}} \int_0^{\eta} e^{-\delta^2} d\delta$$

Therefore:

$$\frac{\Delta C}{C} = \frac{\mathrm{erf}(\eta - \Delta\eta) - \mathrm{erf}(\eta)}{1 - \mathrm{erf}(\eta)} \qquad ; \qquad \Delta\eta \equiv \frac{l_0}{\sqrt{4 \cdot D \cdot t}}$$

For $\eta \ll 1$,

$\mathrm{erf}(\eta) \sim \eta$, and therefore: $\Delta C / C \sim \Delta\eta$

The **lower boundary** of the 'sensing area' (dark gray in Fig. 6B) occurs at short times (t_{min}) (large η) when $C(t)$ becomes larger than C_{min}, or

$$C_0 \cdot [1 - \mathrm{erf}(\eta)] > C_{min} \quad or \quad \eta < erfinv(1 - C_{min}/C_0)$$

However, since C_{min}/C_0 is generally small ($\sim 10^{-7}$), $erfinv(1 - C_{min}/C_0) \sim 1 - 5$
and

$$t_{min} \propto \frac{d^2}{D}$$

The **higher boundary** of the 'sensing area' (dark gray in Fig. 5B) occurs at long times (small η) when $\Delta C/C$ becomes smaller than $\Delta C/C_{min}$.
Generally, $\Delta C/C$ decreases for shorter distances and longer times. Thus, $\Delta C/C$ will first become smaller than $\Delta C/C_{min}$ at short distances and the longest time (t_{max}); the upper- left corner of the plot in Fig. 6B. Thus the point where the "sensing area" starts to decrease, labeled as S_C (Fig. 6C), is given by $\Delta C/C(t_{max}, \eta \to 0) \sim \Delta\eta(t_{max}) \sim \Delta C/C_{min}$, or

$$S_C \sim \frac{l_0}{\sqrt{4 \cdot D \cdot t_{max}}}$$
